# Efficacy of belimumab combined with rituximab in severe systemic lupus erythematosus: study protocol for the phase 3, multicenter, randomized, open-label Synbiose 2 trial

**DOI:** 10.1186/s13063-022-06874-w

**Published:** 2022-11-12

**Authors:** Mieke van Schaik, Eline J. Arends, Darius Soonawala, Ellen van Ommen, Karina de Leeuw, Maarten Limper, Pieter van Paassen, Tom W. J. Huizinga, René E. M. Toes, Cees van Kooten, Joris I. Rotmans, Ton J. Rabelink, Y. K. Onno Teng

**Affiliations:** 1 Center of Expertise for Lupus, Vasculitis and Complement-mediated Systemic disease (LuVaCs), Department of Internal Medicine - Section Nephrology, Leiden University Medical Center, Albinusdreef 2, 2333 ZA Leiden, the Netherlands; 2 Department of Nephrology, HagaZiekenhuis, Els Borst-Eilersplein 275, 2545 AA The Hague, the Netherlands; 3 Department of Nephrology, Radboud University Medical Center, Geert Grooteplein Zuid 10, 6525 GA Nijmegen, the Netherlands; 4 Department of Rheumatology and Clinical Immunology, University Medical Center Groningen, Hanzeplein 1, 9713 GZ Groningen, the Netherlands; 5 Department of Rheumatology and Clinical Immunology, University Medical Center Utrecht, Heidelberglaan 100, 3584 CX Utrecht, the Netherlands; 6 Department of Nephrology and Clinical Immunology, Maastricht University Medical Center, P. Debyelaan 25, 6229 HX Maastricht, the Netherlands; 7 Department of Rheumatology, Leiden University Medical Center, Albinusdreef 2, 2333 ZA Leiden, the Netherlands; 8 Department of Nephrology, Leiden University Medical Center, P.O. Box 9600, 2300 RC Leiden, the Netherlands

**Keywords:** Systemic autoimmune disease, Systemic lupus erythematosus, Lupus nephritis, Belimumab, Rituximab, Clinical trial

## Abstract

**Background:**

Belimumab, an anti-B-cell activating factor antibody, is approved for the treatment of auto-antibody positive systemic lupus erythematosus with a high degree of disease activity. Anti-CD20 B cell depletion with rituximab is used in refractory SLE as well, although with variable responses. We hypothesized that incomplete B cell depletion, related to a surge in BAFF levels following rituximab treatment, can cause ongoing disease activity and flares. The Synbiose 1 study primarily focused on immunological effects and shows the preliminary clinical benefit of combined rituximab and belimumab in SLE. The Synbiose 2 study will evaluate the clinical efficacy of combining belimumab with rituximab in patients with severe SLE, allowing the tapering of prednisolone and mycophenolate.

**Methods:**

Synbiose 2 is a phase 3, multicenter, randomized, controlled, open-label 2-year clinical trial. Seventy adults with severe SLE including lupus nephritis will be randomized 1:1 to receive either standard of care consisting of prednisolone and mycophenolate as induction and maintenance treatment, or belimumab and rituximab combined with standard of care as induction treatment, followed by prednisolone and belimumab as maintenance treatment. The primary objective is to assess whether combined B cell therapy will lead to a reduction of treatment failure. Secondary endpoints are complete and partial clinical and renal response and the improvement of SLE-specific autoimmune phenomena. Safety endpoints include the incidence of adverse events, with a special interest in infections.

**Discussion:**

The Synbiose 2 trial is the first multicenter phase 3 clinical trial investigating combined B cell targeted therapy in SLE, including lupus nephritis. The outcome of this study will provide further evidence for the clinical efficacy of this new treatment strategy in severe SLE.

**Trial registration:**

ClinicalTrials.gov NCT03747159. Registered on 20 November 2018.

## Strengths and limitations of this study

- ◦ This is a randomized, controlled phase 3 trial with 2 years of follow-up, focusing specifically on patients with severe SLE including lupus nephritis
- ◦ This study’s hypothesis was built on a strong scientific basis of preclinical and clinical studies of belimumab and rituximab used as single agents as well as combined in SLE patients
- ◦ The design includes tapering of mycophenolate and prednisolone as maintenance therapy in the intervention group
- ◦ This study compares two different treatment strategies rather than a single add-on, placebo-controlled agent, which could be perceived as a limitation. However, this set-up is closely related to clinical practice making the outcomes directly translatable to patient care
- ◦ The study is limited by its open-label design

## Introduction

Systemic lupus erythematosus (SLE) is a chronic autoimmune disease in which the loss of tolerance to nucleic acids and their binding proteins results in the generation of autoantibodies that initiate and propagate tissue-damaging inflammation involving every organ system. Many patients require life-long immunosuppression, often with high-dose corticosteroids, cyclophosphamide, or mycophenolate mofetil, non-specifically targeting the immune system to reduce inflammation. This results in low-level disease activity in only 25–44% of patients in the long term, while sustained complete remission is rare [1–4]. Moreover, approximately 10% of lupus nephritis (LN) patients develop end-stage renal disease in 5 years, increasing to over 20% in 15 years [5]. Side effects of the current treatment strategies are (opportunistic) infections in the short term and risk for malignancy and cardiovascular disease in the long term, contributing to the reduced life expectancy of SLE patients [6, 7]. This substantiates the need for developing better strategies to prevent and treat the sequelae of SLE.

Treating SLE patients with biologicals is attractive because they have the potential to specifically target key culprits in the pathogenesis of SLE, possibly increasing efficacy and reducing the risk for infections or malignancies as compared to conventional immunosuppressants. Rituximab is a B cell-depleting anti-CD20 monoclonal antibody that has shown efficacy in several retrospective and open-label studies [8–17]. However, two phase 3 double-blind, placebo-controlled trials were unable to meet their primary endpoints, limiting its clinical use to off-label treatment of refractory SLE only [18, 19]. Post hoc analysis in LN patients demonstrated that depth of B cell depletion was associated with complete renal response upon rituximab treatment, suggesting that a more profound B cell depletion yields better clinical outcomes [20]. This premise was corroborated by a recent positive phase 2 study with obinutuzumab, a novel anti-CD20 monoclonal antibody therapy characterized by improved B cell depletion [21].

Another explanation for the variable efficacy of rituximab is its association with elevated levels of B cell activating factor (BAFF, also known as BLyS). BAFF is a cytokine necessary for the survival, activation, and differentiation of B cells to plasma cells and the integrity of germinal centers. BAFF levels are elevated in SLE and are associated with disease activity and flares [22–25]. Upon rituximab-induced B cell depletion, increased BAFF levels are observed which can be capable of triggering a feedback loop of B and T cell activation resulting in a surge of the humoral autoimmune response [26, 27]. In a cohort study, this was reflected by a rise in anti-double-stranded DNA (anti-dsDNA) levels and relapse [28]. These observations suggest that the clinical efficacy of anti-CD20 mediated B-cell depletion can be increased by the addition of a BAFF-inhibiting agent.

Belimumab—a recombinant monoclonal IgG1λ antibody that antagonizes circulating BAFF—was approved as add-on therapy in adults with active, auto-antibody positive SLE with a high degree of disease activity despite standard therapy, after the results of two pivotal phase 3 clinical trials and a subsequent trial for LN [29–31]. Despite showing superior efficacy compared to standard of care, belimumab alone as add-on therapy was not able to achieve more than 30% complete renal response in LN. The Synbiose 1 (*Synergetic B cell immunomodulation in SLE*) study was the first to comprehensively describe the clinical and immunological effects of combining rituximab and belimumab in a cohort of patients with severe, refractory SLE [27, 32, 33]. From this study, we have learned that combining rituximab with belimumab was safe and well-tolerated with clinically relevant positive responses. Briefly, Synbiose 1 demonstrated that long-term belimumab treatment restrained full B cell repopulation leading to long-lasting reduction of anti-dsDNA, anti-C1q and ENA autoantibodies, and neutrophil extracellular traps (NETs). In 67% of highly treatment-refractory patients, clinical response was observed, of which 80% continued belimumab maintenance treatment during the 2-year follow-up, allowing discontinuation of mycophenolate, and tapering of corticosteroids to 7.5mg or less. Subsequently, CALIBRATE and BEAT-LUPUS have conformed in randomized phase 2 studies that the combination of belimumab and rituximab is safe and achieves relevant immunological and clinical endpoints in refractory SLE and LN patients [34, 35]. These studies showed suppressed B cell repopulation and enhanced negative selection of autoreactive B cells, lower anti-dsDNA levels, and reduced risk of severe flares upon combined B cell therapy. Collectively, these studies have prompted us to conduct the Synbiose 2 study, a phase 3 randomized study to investigate the long-term efficacy and safety of belimumab combined with rituximab in severe SLE patients.

## Methods

### Study design

This is a phase 3, multicenter, randomized, controlled, open-label, noninferiority 2-year study conducted in The Netherlands, investigating the efficacy and safety of belimumab combined with rituximab. A list of participating academic and non-academic hospitals can be found on www.clinicaltrials.gov.

### Study population

The study will include adult SLE patients with severe disease with major organ involvement, including LN. In brief, patients are above 18 years of age and have a clinical diagnosis of SLE according to the Systemic Lupus International Collaborating Clinics (SLICC) criteria. Severe disease is defined as either an SLE disease activity index (SLEDAI) ≥ 12, new or progressive activity in major organ systems, or otherwise high disease activity despite conventional immunosuppressive treatment, which requires remission induction therapy. Detailed in- and exclusion criteria are summarized in Table 1.

**Table 1:** Entry criteria. In- and exclusion criteria of the Synbiose 2 study

Inclusion criteria	Exclusion criteria
• ≥ 18 years of age • Having a clinical diagnosis of SLE according to the SLICC criteria 2012 • Severe, active SLE defined as one or more of the following: ◦ SLEDAI ≥ 12 ◦ New or worse SLE-related activity in major organs, i.e., CNS-SLE (includes NPSLE), vasculitis, nephritis, pericarditis and/or myocarditis, myositis, thrombocytopenia (< 60 × 10^9^/L), hemolytic anemia (Hgb < 4.4mmol/L = 7.0 g/dL) ◦ High disease activity that requires or warrants induction treatment by switching to or increasing dosage of oral mycophenolate • New, persisting or progressive disease activity despite the use of conventional maintenance immunosuppressive treatment (e.g., mycophenolate or azathioprine) • Confirmed positive SLE-specific autoantibodies defined as one or more of the following: ◦ ANA titer ≥ 1:80 ◦ Either 2 positive test results from independent time points within the study screening period, or one positive historical test result and 1 positive result during the screening period ◦ Anti-dsDNA serum antibody ≥ 30 IU/mL ◦ Either 2 positive test results from independent time points within the study screening period, or one positive historical test result and 1 positive result during the screening period	• Female patients who arepregnant or breastfeeding • Significant hypogammaglobulinemia (IgG < 4.0 g/L) or IgA deficiency (IgA < 0.1 g/L) • Immunization with a live vaccine within 1 month preceding day 0 • Active infection, defined as one or more of the following: ◦ Hospitalization for treatment of infection within 60 days preceding day 0 ◦ Use of parenteral antimicrobial agents within 60 days preceding day 0 ◦ Serological evidence of viral hepatitis defined as: patients positive for HbsAg or HbcAb, or hepatitis C antibody positivity, not treated with antiviral medication • Having a historically positive HIV test or test positive at screening • Having a history of a primary immunodeficiency • Having a neutrophil count of < 1.5 × 10^9^/L • Having a significant infection history that in the opinion of the investigator makes the candidate unsuitable for the study • Having a history of an anaphylactic reaction to parenteral administration of contrast agents, human or murine proteins or monoclonal antibodies • Having any other clinically significant abnormal laboratory value that in the opinion of the investigator makes the candidate unsuitable for the study • Drug or alcohol abuse or dependence within 1 year preceding day 0 • Having an active malignant neoplasm or one in the last 5 years, except basal cell or squamous cell carcinoma of the skin treated with local resection only, or carcinoma in situ of the uterine cervix treated locally and with no evidence of metastatic disease for 3 years • Having evidence of serious suicide risk, including suicidal behavior in the last 6 months and/or any suicidal ideation in the last 2 months

### Randomization

Seventy patients will be randomized 1:1 to one of two treatment strategies by computer-generated allocation in blocks, to either standard of care, consisting of corticosteroids plus mycophenolate as induction and maintenance therapy, or the intervention group where induction therapy consists of standard of care with the addition of belimumab and rituximab, and maintenance therapy of low-dose prednisolone and belimumab. Enrolment and randomization will be performed by the patient-treating physician researcher or primary investigator of the study center and are done as soon as possible after screening and obtaining informed consent, ideally before initiation of remission induction treatment, with a maximum of 4 weeks thereafter.

### Study treatments

All patients will be given standard of care consisting of 3 pulses of 500 mg (< 60 kg) or 1000mg (> 60 kg) of intravenous methylprednisolone. This will be followed by tapered oral prednisolone at a starting dose of 60mg daily together with mycophenolate at a starting dose of 500mg twice daily and increased weekly, titrated to achieve a target AUC of 60-90mg*h/L, with a maximum dose of 4000mg per day. In cases where therapeutic drug monitoring (TDM) is not possible, mycophenolate dose will be titrated against leukocyte counts and tolerability of the patient. Patients randomized to the intervention group will receive standard of care combined with self-administered subcutaneous belimumab 200mg weekly, and two doses of 1000-mg intravenous rituximab 4 and 6 weeks after belimumab initiation. One hundred-milligram intravenous methylprednisolone, 2-mg intravenous clemastine, and 1000-mg oral acetaminophen will be administered prior to rituximab infusions.

**Fig. 1:**
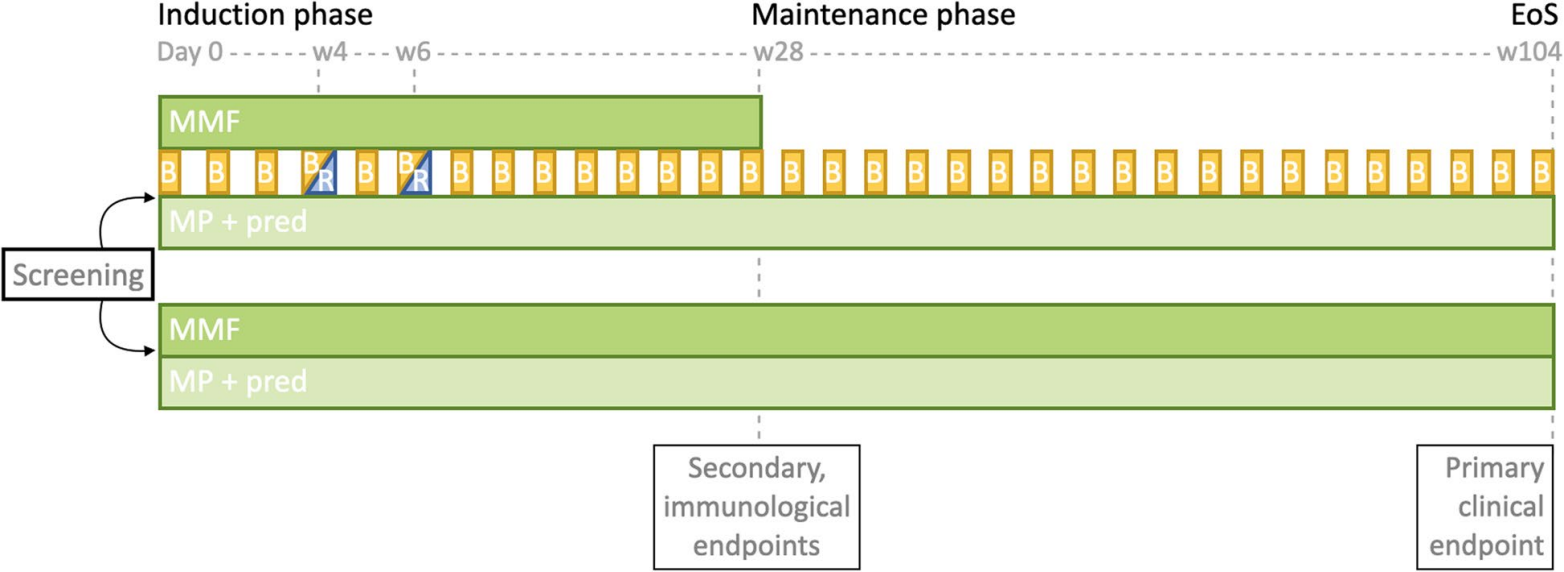
Schematic representation of the study design. MMF, mycophenolate mofetil; MP, methylprednisolone; pred, oral prednisolone; B, belimumab; R, rituximab; EoS, end of study; w, week

In both treatment groups, oral prednisolone will be tapered to 7.5mg before week 28 and to 5mg before week 52 and will be continued at a low dose for the remaining study period. Dose reduction to zero can be personalized to the tolerance of the patient, judged by the treating physician. In the control group, mycophenolate will be reduced by 50% at week 28 and continued for the remainder of the study period. In the intervention group, mycophenolate dose will be reduced by 50% at week 16 and discontinued at week 28. Belimumab will be continued throughout the entire study period. Consequently, after week 28, patients will enter the maintenance phase where they will receive either prednisolone and mycophenolate (control group) or prednisolone and belimumab (intervention group) (Fig. 1). Patients will be monitored closely during the study, with laboratory evaluation and study visits every 2 weeks during the first 8 weeks after inclusion, and increasingly longer intervals during the remainder of the study, up to 2 months eventually. An overview of study visits and assessments per visit is summarized in Table 2.

**Table 2:** SPIRIT figure; overview of all measures

		Study period																				
			Evaluation visits																		EoS	FU
Timepoint (weeks)	**S**	**0**	**B**^**a**^	**2**	**4**	**6**	**8**	**12**	**16**	**22**	**28**	**36**	**44**	**52**	**60**	**68**	**76**	**84**	**92**	**100**	**104**	**116**
**Enrollment**																						
Eligibility screen	x																					
Informed consent	x																					
Allocation		x																				
**Interventions**																						
History	x		x	x	x	x	x	x	x	x	x	x	x	x	x	x	x	x	x	x	x	x
Physical examination	x		x	x	x	x	x	x	x	x	x	x	x	x	x	x	x	x	x	x	x	x
SBLA: hematology	x		x	x	x	x	x	x	x	x	x	x	x	x	x	x	x	x	x	x	x	x
SBLA: immunophenotyping	x		x	x	x	x	x	x	x	x	x		x	x	x	x	x	x	x	x	x	
SBLA: chemistry	x		x	x	x	x	x	x	x	x	x	x	x	x	x	x	x	x	x	x	x	x
SBLA: Ig/complement	x		x	x	x	x	x	x	x	x	x	x	x	x	x	x	x	x	x	x	x	
SBLA: autoantibodies	x		x	x	x	x	x	x	x	x	x	x	x	x	x	x	x	x	x	x	x	
SBLA: virus serology	x																					
Urine analysis + cytology	x		x	x	x	x	x	x	x	x	x	x	x	x	x	x	x	x	x	x	x	x
24h urine analysis	x		x		x			x			x		x		x		x		x		x	
Electrocardiogram	x																					
Chest x-ray	x																					
Kidney biopsy^b^	x																					
Blood product storage	x		x	x	x		x	x	x		x		x		x		x		x		x	
**Assessments**																						
SLEDAI/PGA	x		x	x	x	x	x	x	x	x	x		x	x	x	x	x	x	x	x	x	
SLICC damage score	x														x						x	
QoL questionnaire			x					x			x				x				x		x	
Treatment failure																					x	
Clinical and renal response											x										x	
Autoantibody reduction											x										x	
B cell depletion											x										x	
NET assay			x								x										x	

*S* screening, *B* baseline, *QoL* SF-36 quality of life questionnaire, *FU* follow-up, *SBLA* standard blood laboratory analysis, *Ig* immunoglobulins, *NET* neutrophil extracellular traps

^a^Baseline may be left out if planned within two weeks of screening. ^b^Kidney biopsy only in case of suspected renal involvement

Upon severe flares of SLE disease activity, alternative treatment options will be considered, which can result in the withdrawal of the subject from the study. Patients randomized to the standard-of-care treatment group can cross over to the intervention group in the case of either a severe flare or insufficient treatment response requiring a switch in medication. They will then be considered to have met the primary endpoint of treatment failure, will re-enter the study if they fulfill the inclusion criteria, and receive the complete study treatment of the intervention group as described above.

### Concomitant medication

Cotrimoxazole, antimalarials, intra-articular injections, NSAIDs, and statins can be initiated or continued during the study. Antiproteinuric agents are allowed at the discretion of the treating physician, according to the national guidelines.

Anti-TNF therapy, cyclophosphamide, and biologicals other than belimumab and rituximab are prohibited at any time during the study period and in the 60 days prior to day 0. Live vaccines are prohibited within 30 days prior to day 0. Any combination of mycophenolate with the following is not allowed: methotrexate, azathioprine, leflunomide, calcineurin inhibitors, sirolimus, 6-mercaptopurine, and thalidomide. Finally, any investigational agent outside of this study is prohibited.

### Study endpoints

The primary clinical endpoint is the proportion of patients with treatment failure during the trial period, defined as either death, development of end-stage renal disease, major renal or extrarenal flare, sustained doubling of serum creatinine, clinical or renal non-response, requiring induction therapy or plasmapheresis, requiring a dose reduction or discontinuation of mycophenolate—other than established by protocol—for more than 14 or 7 days respectively, discontinuation of belimumab for more than three consecutive doses, inability to complete two rituximab administrations, withdrawal for any reason, or loss to follow-up.

Secondary clinical endpoints will be evaluated at 28 and 104 weeks. Clinical response is defined as a reduction in SLEDAI of ≥ 4 points and no worsening of Physician Global Assessment (PGA) score of > 0.3 points on a scale of 0–3. Other secondary endpoints are clinical remission (defined as meeting the criteria of clinical response and additionally achieving to taper steroids to ≤ 5 mg/day), renal response (complete renal response being < 0,75gr proteinuria per 24 h and a serum creatinine within 125% of baseline), low-level disease activity (SLEDAI ≤ 4 with no activity in major organ systems, hemolytic anemia or gastrointestinal activity and no new disease, PGA ≤ 1 and ≤ 7.5mg prednisolone equivalent), and disease control (SLEDAI ≤ 2 with ≤ 5mg steroids per day) [36]. Patient-reported outcome measure includes the SF-36 Quality of Life questionnaire [37]. Other clinical secondary endpoints are feasibility and safety of combination treatment by monitoring the occurrence of adverse events, with special attention to infections.

The main immunological endpoint is reduction of anti-dsDNA and other disease-specific autoantibodies at 28 weeks, which is also assessed at 104 weeks. Secondary immunological endpoints are reduction of relevant autoantibodies, sustained B cell depletion, and regression of immune complex-mediated excessive NET production at 28 and 104 weeks.

### Sample size calculation

The power analysis to determine sample size of the study was based on data from the Synbiose 1 trial (i.e., combination treatment with belimumab and rituximab) and the ALMS trial (i.e., standard of care with mycophenolate and steroids) [32, 38]. To show a significant reduction in treatment failure, we assumed a treatment failure rate during the study period of 104 weeks of 54% in the standard of care group (in accordance with ALMS study results) compared to 23% in the intervention group (in accordance with the Synbiose 1 study results). We calculated that in order to demonstrate a hazard ratio of 2.97 with at least 80% power, using an alpha error confidence level of 5% and beta probability of 0.20, the study would require a total of 70 patients to be randomized with 35 patients in each group.

### Statistical analysis

The statistical analysis will primarily be performed on the intention-to-treat population. For non-biased immunological endpoints, a per-protocol analysis will be done to assess the pathophysiological effects of adding belimumab and rituximab onto standard of care. The observed rates of treatment failure will be displayed in survival curves and the difference between treatment groups will be assessed using a log-rank test.

Differences in baseline characteristics, immunological endpoints, and clinical response will be explored. The absolute and percentage reduction of autoantibody titers, memory B cells, and NET formation will be analyzed as continuous variables using the non-parametric Mann-Whitney *U* test to compare treatment groups. Clinical disease activity categorized by the aforementioned response criteria will be analyzed using the chi-square test for dichotomized variables. Dosages of immunosuppressants will be analyzed as continuous variables using Mann-Whitney *U* because of the limited number of patients. Missing data will be imputed as that subject’s previously observed value. Patients that have crossed over from the control group to the intervention group will be considered as having met the primary endpoint of treatment failure and will re-enter the study as a new subject. As such, they will be included in the intention-to-treat analysis. Also, they will serve as their own controls in an additional post hoc analysis.

Safety, toxicity, and adverse events will be documented, and the events considered to be at least possibly related to the study treatment will be summarized by severity and treatment group. Infectious AE’s will be presented to an independent infectious disease specialist for evaluation of severity and relation to study participation.

### Ethical considerations and dissemination

This investigator-initiated study was reviewed and approved by the Medical Ethics Committee Leiden-The Hague-Delft and registered at www.clinicaltrials.gov (NCT03747159). All participating sites will additionally be reviewed for site competency and suitability by their local executive board upon initiation. The trial will be conducted according to the International Conference of Harmonization - Good Clinical Practice quality standard and all other applicable regulatory requirements and adheres to the ethical principles that have their origin in the Declaration of Helsinki. The coordinating center will be responsible for overall trial and data management, monitoring and communication among all sites, and general oversight of the conduct of the trial. The steering committee will consist of the primary investigators of the coordinating and participating centers. They will provide overall supervision and ensure that the trial is conducted to the abovementioned standards.

Written informed consent will be obtained from all patients prior to the initiation of any study-specific procedure or data collection and includes consent for the use of participant data and specimens in potential follow-up studies. All data is stored in a standardized manner in an electronic data capture platform with restricted access. Data integrity is enforced through the following mechanisms. All data entry is carried out by a dedicated member of the research team. Source data will be kept on file at the participating sites and consistency checks against data stored in the database will be done at regularly scheduled monitoring visits. Documentation of changes will be available via audit trails. Patient privacy is ensured by de-identifying all data submitted to the electronic data capture platform and using subject identification codes. The chief investigator will have access to all data, and the other primary investigators will have access to the trial datasets of patients participating in their own medical centers. All patients will have the right to withdraw from the study at any time during the trial. It is of note that the possibility of crossing over in the case of a severe flare or insufficient response to standard of care increases the advantage of participation for patients, because this makes them eligible for a novel treatment strategy in a situation where treatment options may otherwise be limited. Follow-up evaluations will be performed for all patients at least 3 months after completing the 2-year follow-up and once a year thereafter if possible.

Monitoring will take place two times a year and the monitoring process will be independent from the investigators. Auditing visits will take place as deemed necessary by the auditing committee. Severe adverse events will be reported to the sponsor within 24 h. A data monitoring committee was not necessary based on the risks of the study. However, to ensure subject safety, safety meetings with independent specialists will be held four times a year in order to monitor and evaluate adverse events, treatment failures, and drop-out. A clinical trial insurance that provides coverage for damage to research subjects through harm caused by the study is available in accordance with the legal requirements in The Netherlands. When medically indicated, trial visits can be intensified as desired or supplemented with the trial’s independent physician in the case of adverse events or harm that occurred during the trial. Additional health care needs that arise during or as a consequence of the trial will be adequately addressed by the investigators.

Several methods are employed to improve participant retention and completion of follow-up, minimizing the burden and practical implications for patients. Scheduling of appointments is planned well ahead of time and reminders are included. Travel allowance will be provided for unplanned visits to the study center. Furthermore, patients who are withdrawn from the study medication will be followed at regularly scheduled study visits as specified by the protocol, unless informed consent is withdrawn. Treatment during the study is defined up to 104 weeks, after which the treatment with belimumab can be continued or patients can be switched to another immunosuppressive regimen as preferred by the treating physician in shared decision with the patient. In the Netherlands, belimumab is covered by insurance companies for the treatment of LN patients posing no barrier to continue any medication from either treatment arm in routine clinical practice after completion of the study.

Significant protocol amendments will be communicated with all relevant parties. We will submit our findings for peer-reviewed, open-access publication, and data resulted from this randomized clinical trial as well as other related documentation will be available on reasonable request. This paper complies with the Standard Protocol Items: Recommendations for Interventional Trials (SPIRIT) recommendations for protocol reporting [39].

### Patient and public involvement

Patients and public were not involved in the development of this study.

## Discussion

This investigator-initiated trial was developed through analysis of clinical and immunological data of SLE patients using a combination treatment with rituximab and belimumab, a targeted and immunologically synergistic treatment for which there is great promise in autoantibody-mediated disease such as SLE and LN. The Synbiose 2 study will compare efficacy of two treatment strategies—standard of care alone versus standard of care and additional belimumab and rituximab—by assessing treatment failure over the study period of 2 years. Subsequently, the study will allow investigation of the potential improvement of targeting autoreactive B cells by starting treatment with subcutaneous belimumab followed by rituximab, and study the immunological effects on B-cell depletion and repopulation, circulating autoantibodies and NET formation. Ultimately, by evaluating the ability to reduce cumulative steroid dose and rapidly tapering mycophenolate while avoiding a flare of disease activity, this study addresses the clinical problem of long-term immunosuppressant toxicity as well as specifically targeting the humoral autoimmune responses in SLE patients. As such, the use of combined B cell targeted therapy may be thus be supported even in the case of equal efficacy to established treatment strategies.

An important feature in the Synbiose 2 study design is the initiation of belimumab before rituximab. The order of initiation of each agent is very relevant from an immunological perspective, for several reasons. Firstly, rituximab effectively and nearly completely depletes circulating B cells. However, a significant amount of resistant B cells reside in lymphoid tissues such as bone marrow, lymph nodes, and inflamed tissues [40–42]. Consequently, we have observed in the Synbiose 1 study—where belimumab was introduced *after* rituximab—that repopulation of immature B cells was prevented by belimumab and early repopulation was dominated by memory B cells and plasma cells that likely had been resistant to depletion by rituximab. Secondly, a significant but unexpected surge in circulating memory B cells has been described in several studies in the first weeks after belimumab add-on therapy [43–46]. A recent study has corroborated this phenomenon and identified that belimumab disrupts the trafficking of memory B cells [47]. These observations laid the foundation of the Synbiose 2 study design, which intends to benefit from the surge of memory B cells in the circulation by belimumab, which are then susceptible to depletion by rituximab.

In the study design, TDM of mycophenolate was included in order to ensure an optimal immunosuppressive regimen in both groups. Adequate TDM is important not only for a fair comparison of treatment regimens with respect to immunological outcome parameters but also in light of the possibility in this study to cross over. Crossing over to treatment with belimumab and rituximab is intended for relapsing patients in the control group in case of insufficient treatment response or a severe flare while on adequate immunosuppression, which is ensured by employing TDM. It is important to emphasize that the option to cross over allows the inclusion of relapsing patients who might otherwise have limited therapeutic options. Of note, patients that cross over will provide additional information by allowing an intra-individual comparison by serving as their own control in a post hoc analysis. In order to avoid cumulative over-suppression of the immune system in the intervention group, mycophenolate dose will be quicky tapered and discontinued at the onset of the maintenance phase, although it can be hypothesized that its cytotoxic effect on B-cells will already be abated in the case of deeper B-cell depletion.

In conclusion, the Synbiose 2 study builds on previous phase 2 studies confirming safety and preliminary evidence of clinical benefit of combined B cell-targeted therapy in SLE [32, 34, 35]. The Synbiose 2 study focuses on severe SLE and LN patients which is complementary to the phase 3, randomized BLISS-BELIEVE study (NCT03312907)—which investigates a similar treatment regimen in non-renal SLE. The outcome of this study will provide further evidence for a new treatment strategy in severe SLE and potentially challenge the current restriction in the belimumab label that warns against combining with another B-cell-depleting agent [48].

### Trial status

The trial began recruitment in November 2018 and is estimated to be complete in September 2025. The current protocol version is 10 (April 4, 2022).

## Data Availability

The results of this trial will be submitted for publication in a peer-reviewed open-access journal. The writing committee will consist of the authors of this manuscript and all primary investigators of the medical centers that are initiated to participate in the trial. The resulting data of the trial will be available upon reasonable request after publication of the major findings. A model consent form has been provided as a supplement to the manuscript. Additional documents (such as study protocol) may be available.

## Abbreviations

Anti-dsDNA: Anti-double-stranded DNA
BAFF: B cell activating factor
LN: Lupus nephritis
NET: Neutrophil extracellular trap
PGA: Physician global assessment
SLE: Systemic lupus erythematosus
SLEDAI: Systemic lupus erythematosus disease activity index
SLICC: Systemic lupus international collaborating clinics
TDM: Therapeutic drug monitoring
